# Barriers and Facilitators to Implementation of Value-Based Care Models in New Medicaid Accountable Care Organizations in Massachusetts: A Study Protocol

**DOI:** 10.3389/fpubh.2021.645665

**Published:** 2021-04-06

**Authors:** Sarah L. Goff, Deborah Gurewich, Matthew Alcusky, Aparna G. Kachoria, Joanne Nicholson, Jay Himmelstein

**Affiliations:** ^1^Department of Health Promotion and Policy, University of Massachusetts, Amherst, MA, United States; ^2^Center for Healthcare Organizations and Implementation Research, US Department of Veterans Affairs, Boston, MA, United States; ^3^Population and Quantitative Health Sciences, University of Massachusetts Medical School, Worcester, MA, United States; ^4^Commonwealth Medicine, University of Massachusetts Medical School, Shrewsbury, MA, United States; ^5^Heller School for Policy and Management, Brandeis University, Waltham, MA, United States

**Keywords:** value-based care, accountable care organization, implementation, Medicaid, disparities

## Abstract

**Introduction:** Massachusetts established 17 new Medicaid accountable care organizations (ACOs) and 24 affiliated Community Partners (CPs) in 2018 as part of a large-scale healthcare reform effort to improve care value. The new ACOs will receive $1.8 billion dollars in state and federal funding over 5 years through the Delivery System Reform Incentive Program (DSRIP). The multi-faceted study described in this protocol aims to address gaps in knowledge about Medicaid ACOs' impact on healthcare value by identifying barriers and facilitators to implementation and sustainment of the DSRIP-funded programs.

**Methods and analysis:** The study's four components are: (1) Document Review to characterize the ACOs and CPs; (2) Semi-structured Key Informant Interviews (KII) with ACO and CP leadership, state-level Medicaid administrators, and patients; (3) Site visits with selected ACOs and CPs; and (4) Surveys of ACO clinical teams and CP staff. The Consolidated Framework for Implementation Research's (CFIR) serves as the study's conceptual framework; its versatile menu of constructs, arranged across five domains (Intervention Characteristics, Inner Setting, Outer Setting, Characteristics of Individuals, and Processes) guides identification of barriers and facilitators across multiple organizational contexts. For example, KII interview guides focus on understanding how Inner and Outer Setting factors may impact implementation. Document Review analysis includes extraction and synthesis of ACO-specific DSRIP-funded programs (i.e., Intervention Characteristics); KIIs and site visit data will be qualitatively analyzed using thematic analytic techniques; surveys will be analyzed using descriptive statistics (e.g., counts, frequencies, means, and standard deviations).

**Discussion:** Understanding barriers and facilitators to implementing and sustaining Medicaid ACOs with varied organizational structures will provide critical context for understanding the overall impact of the Medicaid ACO experiment in Massachusetts. It will also provide important insights for other states considering the ACO model for their Medicaid programs.

**Ethics and dissemination:** IRB determinations were that the overall study did not constitute human subjects research and that each phase of primary data collection should be submitted for IRB review and approval. Study results will be disseminated through traditional channels such as peer reviewed journals, through publicly available reports on the mass.gov website; and directly to key stakeholders in ACO and CP leadership.

## Contributions to the Literature

- This study will be one of the first to systematically identify barriers and facilitators to implementing and sustaining a large-scale systems transformation initiative across the duration of the 5-year program.
- Understanding implementation barriers and facilitators will provide important context for interpreting the overall impact of the systems transformation initiative on quality and costs of care.
- Findings of the study are expected to have utility for policy-makers and health system leaders considering implementation of innovative health care delivery models in the U.S. and abroad.

## Introduction

Global efforts to address the rising costs of healthcare while maintaining or improving quality of care have increasingly included implementation of accountable care organizations (ACOs) (1–3). The value-based payment models used by ACOs incentivize quality of care and cost reduction through payer-provider partnerships in which financial risks are shared. The ACO model aligns financial incentives with improvements in care integration and coordination across health and social service sectors (4), differing from fee-for-service payment models' prioritization of volume and intensity of care. This important shift in financial incentives prioritizes prevention and population health, which has the potential to improve healthcare delivery and clinical outcomes for patients at higher risk for experiencing healthcare inequities and health disparities.

The majority of research on ACOs to date in the U.S. has focused on changes in quality of care, costs, and patient outcomes associated with Medicare and commercial ACO programs (5–17) in the Medicare system, which insures patients age 65 year of age or older. Identifying and understanding the barriers and facilitators to implementing and sustaining the changes in healthcare delivery encountered by ACOs from the early stages of their inception is critical for interpreting the downstream effects on health care value and patient outcomes. However, prior studies of ACO implementation have focused on Medicare ACOs or been limited to a narrow timeframe or scope (18–20) and none to our knowledge have examined barriers and facilitators to long-term sustainment over time.

The state of Massachusetts's (MA) Medicaid program (MassHealth) contracted with 17 new ACOs and 27 associated Community Partner (CP) organizations in 2018 as part of a 5-year experimental demonstration project, subsequently referred to as the Demonstration. The CPs, an innovative feature of the MA model, work with the ACOs to coordinate and manage care for ACO patients with behavioral health diagnoses or for those who need long-term services and supports (LTSS). This study aims to identify barriers and facilitators to implementation and sustainment of interventions funded by the Delivery System Reform Incentive Payment (DSRIP) program across these new Medicaid ACOs and CPs. The protocol for studying the MA Medicaid ACO experiment's effectiveness at improving quality while maintaining or reducing costs will be reported elsewhere.

## Methods and Analysis

### Overview

This study aims to determine the extent to which the organizations that comprise the ACOs and CPs are able to implement the system transformation initiative as intended and to identify facilitators and barriers to implementation and sustainment. The study has two primary aims: to use a mixed-methods, developmental approach to identify issues with implementation early in the ACO experiment so that adaptations may be made if indicated (21) and to produce generalizable knowledge for federal policy-makers and healthcare systems seeking to transform how healthcare is delivered and supported to vulnerable populations.

The study's theoretical framework draws on the Consolidated Framework for Implementation Research (CFIR) (22). CFIR was chosen as the theoretical framework for the study because its domains (Intervention Characteristics, Outer Setting, Inner Setting, Characteristics of Individuals, and Process of Implementation) and the constructs within the domains are pertinent to studying the implementation and sustainment of complex interventions, such as healthcare delivery transformation. CFIR is also an appropriate framework for this study because its flexible structure is designed to be used across multiple phases of a study, from design through dissemination. Each of the five domains is explored in at least one of the study's four phases.

### ACOs and the Delivery System Reform Incentive Program (DSRIP)

Accountable care organizations (ACOs) are networks of doctors and hospitals that share financial and medical responsibility for providing coordinated care to patients in hopes of limiting unnecessary spending, meaning they aim to increase the value of the care provided (23). The Patient Protection and Affordable Care Act of 2010 made provisions for Medicare, which insures nearly all people age 65 or older in the U.S., to implement ACOs in its program (24, 25). DSRIP funds “support the restructuring of MassHealth's delivery system to promote integrated, coordinated care and hold providers accountable for quality and total cost of care (26).” DSRIP funding in MA is a one-time federal investment of $1.8 billion dollars that phases down over the course of 5 years, after which the programs are expected to be self-sustainable. DSRIP funds pay for programs that support health care delivery transformation in the ACOs and CPs; DSRIP funds also support the MA Statewide Investment program (SWI), which funds activities related to workforce development and retention, technical assistance, enhanced diversionary behavioral health activities, and increasing access for patients with disabilities or for whom English is not the first language (26). Each ACO and CP developed a unique plan to use the DSRIP funds that was tailored to their implementation and sustainment needs and to meet the needs of the patient population they serve (27). The activities funded by the DSRIP program support the ACOs and CPs in achieving the goal of increased value of the care delivered.

### Methods

To achieve the study's aim, implementation and sustainment data will be collected in four-phases: (1) Document Review to characterize ACOs and CPs; (2) Key Informant Interviews (KIIs) with ACOs' and CPs' leadership as well as MassHealth patients in two waves; (3) Site visits conducted with select ACOs and CPs; and (4) Surveys of ACO clinical team members and CP staff. The methods and the analytic plan for each phase are described in detail below; the timeline and goals of each phase of the study are outlined in Table 1. The Institutional Review Board (IRB) at the research team's institution requested to review the procedures for each phase of data collection sequentially. To date, the IRB has determined that Phases 1 and 2 do not constitute human subjects research. Phases 3 and 4 will be submitted prior to beginning these phases and IRB determinations followed.

**Table 1:** Data sources and timeline.

	**Year 1–2**	**Year 3**	**Year 4**	**Year 5**
Document review	✓	✓	✓	✓
State interviews		✓		✓
ACO, CP, and MCO interviews	✓		✓	
Consumer interviews	✓		✓	
Provider and staff survey		✓		✓
ACO and CP site visits		✓		✓

#### Systematic Characterization of the MA ACOs and CPs

The design of the Demonstration gave ACOs and the CPs flexibility in determining their organizational structures and how they plan to utilize DSRIP funding. Given this heterogeneity, we will systematically characterize the organizational structures of each ACO and CP, patient population, budget for DSRIP funds, and plans for implementing DSRIP-funded programs in Phase 1. This will be achieved through extraction of pertinent data from the Participation Plans submitted by each ACO and CP prior to being approved to participate in the Demonstration. The Participation Plans include ACOs' and CPs plans for their governing structures, a description of their patient population, and plans for DSRIP implementation. The Participation Plan data describes how each organization intends to change healthcare delivery within their organization, such as by using health information technology to address health-related social needs and hiring community health workers to support care coordination and management. Data elements to be extracted were determined using the CFIR framework: Intervention Characteristics (specific plans for use of DSRIP funds); Outer Setting (population characteristics), and Inner Setting (governance structures, partnerships/networks, and prior experience with value-based care models). The extracted data will be summarized in streamlined reports that provide systematic categorization of the ACOS and CPs. The reports will be made available to the research team members conducting the KIIs (See section methods and analysis) to enable them to tailor interview questions to pertinent aspects of each ACO's or CP's unique organizational structure and plans for DSRIP spending and implementation of DSRP-funded activities and programs.

#### Key Informant Interviews (KII)

##### Overview

Two waves of semi-structured in-depth interviews will be conducted with representatives of four stakeholder groups: (1) ACOs; (2) CPs; (3) MassHealth staff responsible for administering the DSRIP program, and (4) MassHealth patients. Sample sizes for each group are intended to strike a balance between breadth and depth and to achieve theoretical saturation (no new concepts emerging over three sequential interviews) while minimizing respondent burden (Table 2). Interviews will be conducted with each stakeholder group at interim and end-points of the Demonstration; efforts will be made to interview the same participants in each wave to reduce the chance that any changes reported may be more reflective of change in participant rather than change in implementation processes.

**Table 2:** Sample sizes for study procedures.

	**Years 1–2**	**Year 3**	**Year 4**	**Year 5**
KII MassHealth Leaders		*N* = 10		*N* = 10
KII ACOs (2–3 reps per ACO at 17 ACOs)	*N* = 34–51		*N* = 34–51	
KII CPs (1–2 reps per CP at 27 CPs)	*N* = 27–54		*N* = 27–54	
KII Patients	*N* = 30		*N* = 30	
Provider staff survey		TBD		TBD
ACO site visits for case studies		4 sites		4 sites
CP site visits for case studies		4 sites		4 sites
KII - Key Informant Interview				

##### Sampling and Recruitment

ACOs and CPs will be notified by MassHealth that the research team will be reaching out to invite them to participate in the interviews. The research team will then send a standardized introductory e-mail to the contact listed on the ACO's or CP's Participation Plan document. The e-mail will briefly explain the goals of the KIIs and will ask the contact to identify appropriate key informants in their organization; the e-mail will include an attachment with a synopsis of the study. The research team will then contact the key informants identified by the ACO or CP contact *via* e-mail to address any questions and to schedule the interview.

A Senior Manager at MassHealth will provide the research team with contact information for MassHealth leaders responsible for administering the DSRIP program. These representatives will be invited to participate in an interview *via* e-mail. Sampling will aim to achieve a breadth of experience among those administering the DSRIP program.

For interviews with MassHealth patients, MassHealth leadership will inform contacts at the ACOs and CPs that the research team will be reaching out to them to identify patients who may be willing to share their experiences with changes in healthcare related to the DSRIP program. To understand the needs of as many patients as possible, the research team will review the nominations and purposively recruit patients who are most likely to have experienced changes in healthcare delivery related to the DSRIP program due to the following conditions: (1) medical complexity (multiple medical conditions, which may involve multiple medications and/or high utilization of medical care); (2) living with a disability; (3) receipt of LTSS and/or behavioral health services through a CP; there will be an emphasis on recruiting patients with substance use disorders (SUD); and (4) parents of children utilizing MassHealth (Table 3). Patients who have conditions or life situations that place them in multiple categories (i.e., medical complexity and raising a MassHealth pediatric patient) will be recruited based on one of the target conditions.

**Table 3:** Sampling strategy for patient key informant interviews.

**Patient category**	**Number of interviewees**	**Percent**
Medically complex	10	33.3%
Patients with disabilities	5	16.7%
Pediatric patients	5	16.7%
CP-BH	6	20%
CP-LTSS	4	13.3%

##### Interviews

Interviews will be conducted by trained research staff using semi-structured interview guides tailored to the interview population (Appendix B). Interview questions were developed using the CFIR framework as a guide and include questions pertaining to Outer Setting, Inner Setting. The interviewer will review a study fact sheet with the participant and answer questions prior to beginning the interview. The interview guides for ACO and CP leadership were developed by the research team and pilot-tested with one ACO and one CP. For the patient interviews, an external stakeholder with extensive experience in this arena reviewed the guides for accessibility. Interviews are expected to last ~60–90 min and will be audio recorded and professionally transcribed.

Interview questions for ACO and CP leaders will elicit perspectives on state actions to support delivery system transformation and the effectiveness of these actions. The interviews will aim to understand the factors that facilitate and impede organizational transformation in relation to three CFIR domains: Inner Setting, Outer Setting, and Process. For example, ACO and CP leaders will be asked how prior experience with other value-based payment models informed early stages of implementation in their organization (Inner Setting) and what, if any, factors external to the organization and the DSRIP program they feel may have facilitated or hindered implementation of DSRIP activities or may facilitate or hinder sustainment (Outer Setting).

Interviews with MassHealth leaders will similarly focus on implementation of the DSRIP program, but given their high-level administrative roles, interview questions will also explore implementation of the DSRIP program in the broader context of program and policy implications for the future. Interview methods will otherwise follow those described for ACO and CP interviews.

Interviews with MassHealth patients and caregivers of pediatric MassHealth patients will be conducted *via* telephone and will include questions relative to Outer and Inner Settings in the CFIR framework. To ensure patient interest, accessibility requirements, and understanding, an initial outreach call will be made to inform patients about the project, determine if there are any barriers, such as language or disability, and schedule a time to conduct the interview. Efforts will be made to involve translation services or to accommodate other needs as they arise. Patients will be given a $50 gift certificate as a thank you for their time and candor.

##### Analysis

Interview data will be analyzed using framework analysis (28, 29) and will focus on barriers and facilitators to implementation and sustainment within the three CFIR domains that were the focus of interview questions. We will first establish Interrater reliability among coders (through a process of concurrent open coding of an initial set of interviews, comparison of coding approaches, and refinement of code definitions as needed); the remaining interviews will be coded independently by patients of the analytic team. Once all interview data are coded, secondary coding (combining codes and creating sub-codes) will be performed and analytic matrices with the final coded data created to facilitate across- and within-stakeholder group analysis with respect to perceptions of state actions supporting delivery system transformation, barriers and facilitators to care, and the overall patient experience. Dedoose software (30) will be used to manage, code and analyze interview data, and calculate Cohen's Kappa coefficients (31) to monitor agreement among coders over time.

#### Case Studies

##### Overview

Two waves of site visits will be conducted with a subsample of ACOs and CPs at interim and end-points of the Demonstration to inform case studies (Table 1). The first and second wave of site visits will aim to achieve a deeper understanding of the specific healthcare delivery system innovations that ACOs and CPs are implementing and the contextual factors that may be facilitating or impeding implementation of DSRIP-funded activities and programs (32). The second wave of site visits will also seek to achieve a thorough understanding of facilitators and impediments to sustainability of the ACO and CP models after the end of the DSRIP program.

##### ACO and CP Sampling

In the first wave of site visits, the research team will examine up to four ACOs and four CPs that have achieved different levels of success in transforming care delivery for their MassHealth patients. ACOs and CPs will be selected based on a combination of: (1) their progress in implementing DSRIP-funded projects and (2) differences in organizational structure and populations served. The timing of the site visits will be determined by what is learned from the other data sources with respect to the two dimensions sampling is based upon. For instance, if the research team is able to identify ACOs and/or CPs that excel on implementation of the DSRIP program or are struggling by the 2nd year of the Demonstration, each could be the subject of a site visit. At the same time, it may take until Year 3 of the Demonstration for such patterns to emerge. In sum, we will conduct up to eight site visits between Year 2 and 3 of the study (Table 1). The second wave of site visits will take place in Year 5; up to four ACOs and four CPs that represent higher and lower levels of performance as defined by level of change and/or achievement related to accountability scores being used by MassHealth to determine shared risk payments. For both waves, the site visits will focus on the healthcare delivery transformation activities related to DSRIP that the ACOs and CPs have initiated and the barriers and facilitators to effective implementation, performance, and sustainability.

##### Site Visit Procedures and Case Study Development

Semi-structured interviews and focus groups will be conducted with front-line clinical team members, including providers and staff, who are closely involved with DSRIP implementation and who represent a range of functional roles. Participants in site visit interviews and focus groups will differ from participants in the KIIs, focusing on those responsible for coordinating and delivering clinical care at the ACO and CP practice sites. Participants will include: (1) clinical leads (e.g., medical directors and nurse managers); (2) operational leads (e.g., office managers); (3) heads of health information technology (HIT)/health information exchange (HIE); (4) heads of quality improvement; and (5) heads of support services such as case management. In addition, we will interview representatives of ACO governing boards, Patient and Family Advisory Committees, and selected CPs. At CPs, interviews will be conducted with the following functional roles: (1) clinical leads; (2) administrative directors of CP programs; (3) heads of Health Information Technology/Health Information Exchange. Interview guides will cover similar topics/CFIR domains to those used in key informant interviews with leaders, but will explore more pragmatic aspects of implementation experienced by front-line providers and staff, including constructs in the Characteristics of Individuals domain.

##### Analysis

Analysis of semi-structured interview and focus group data will follow the process described for KIIs to construct a case study for each site (32). In addition, the site visit data will be triangulated with data collected Phases 1, 2, and 4 to compare and contrast perspectives of those in different roles within the ACO and to explore how the site visit data confirm or conflict with related data from other sources.

#### Survey of Front-Line Providers and Staff at ACOs and CPs

To understand how a large sample of front-line providers and staff (e.g., community health workers, social workers, MDs, DOs, NPs, PAs, nurses) experience changes in care delivery related to the DSRIP program, two waves of front-line provider and staff surveys will be conducted at interim and endpoints of the Demonstration. Surveys will aim to assess the degree to which implemented projects and ACO/CP formation are translating into changes in care delivery from the perspective of front-line ACO providers and CP staff. The survey will provide an opportunity to quantitatively measure and compare these experiences between groups of providers, practice types, and ACOs that differ in important characteristics.

##### Questionnaire Development

The questionnaire used for the survey will incorporate the previously validated measures of perceptions of care integration from the Provider and Staff Perceptions of Integrated Care (PSPICs) (33) and new questions developed and pilot tested by the research team to address specific aspects of implementation of the DSRIP program. CFIR domains explored will include Inner Setting (care coordination within the practice site and with external providers and community resources); Outer Setting (patient engagement, MassHealth policies and processes, payment and financial incentives) and Processes (practice site structures and processes). The questionnaire will be pilot tested with a convenience sample of ~10–15 ACO providers and 5–10 CP staff with similar roles to those to be included in the survey sample. Pilot testing will include cognitive testing and assessments for clarity, completeness, and respondent burden.

##### Sampling and Administration

The sampling frame for the ACO provider survey will include providers practicing at group practices, community health centers, and hospital licensed health centers participating in the ACO program at the time the program launched (i.e., 2018). Providers at solo physician practices, outpatient hospitals, practice sites located outside of Massachusetts, sites with fewer than 50 MassHealth patients, and sites with an unknown number of MassHealth patients will be excluded. From within the sampling frame, up to 30 practice sites per ACO (including all sites for those with <30 sites and a random sample of practice for ACOs more than 30 practice sites), thereby oversampling the ACOs with fewer practice sites. The providers practicing at the 353 unique practice sites in this sample will constitute the sample frame for the survey of ACO frontline providers.

The research team will collect provider contact information from practice and ACO administrators. The questionnaire will then be emailed to a random sample of eligible providers (MDs, DOs, NPs, PAs, RNs, LPNs, and LCSWs) for each ACO. Stratified random sampling is expected and the sampling fraction will vary by ACO and provider type such that less prevalent characteristics are oversampled. The contact information for CP staff will be collected from administrators at all 27 CPs and the questionnaire will be emailed to a random sample of staff. As with the ACOs, stratified random sampling is expected and the sampling fraction may vary by CP staff roles such that less prevalent roles are oversampled. The required sample size will be determined based on anticipated response rates and power calculations performed prior to random selection of providers and staff.

##### Analysis

The results of each survey wave will be analyzed overall, by ACO characteristics, practice site characteristics, and by provider/staff characteristics to explore heterogeneity in provider/staff perspectives of the ACO and CP programs. Changes over time between wave one and wave two of the survey will also be examined overall, by ACO characteristics, practice site characteristics, and by provider/staff characteristics. Findings from the survey will be used to measure provider/staff understanding of the ACO and CP programs, their perceived effectiveness, and the concordance of perceptions between front-line providers/staff and their organizational leaders. Findings will also be used to assess the relationship between providers' perceived experience of transformation and ACO/CP care quality and cost performance. In addition to crude analyses, sampling and non-response weights will be applied to obtain estimates that are adjusted for the multi-stage sampling approach and observed sources of non-response bias.

## Discussion

This will be one of the first in-depth longitudinal studies of barriers and facilitators to implementation and sustainment of a large scale, policy-driven, state and federal government funded intervention that aims to improve healthcare value for a vulnerable population of publicly-insured patients. The patient population served by the new Medicaid ACOs in MA is a experiences socioeconomic and racial/ethnic health disparities and healthcare inequities that are not addressed well in traditional models of healthcare delivery in the U.S. The shared risk ACO model, which incentivizes increased value, has the potential to transform healthcare delivery to better address the complexity of social determinants of health within the healthcare system.

The Demonstration is a natural experiment; as such, the DSRIP-supported interventions to facilitate implementation and sustainment of the new ACOs are taking place in uncontrolled settings. Although this limits the capacity to directly compare specific strategies for implementing and sustaining the transformations each ACO and CP undertakes, it also allows for in-depth study of the implementation in a real-world setting. This study is expected to offer important insights into the mechanisms of transforming healthcare delivery and finance to meet the complex medical and social needs of patients in health disparity populations.

## Ethics and Dissemination

### Ethics

The Institutional Review Board (IRB) at the investigative team's institution determined that the overall study did not constitute human subjects research. Each phase of primary data collection will be submitted for IRB review and approved protocols adhered to. Because the investigation is part of a federally mandated evaluation of a state-led intervention, reports of the investigation will ultimately be made available to the public. This level of transparency reinforces the need to ensure that all data be reported in aggregate and ensure that any individuals will not be identifiable.

### Dissemination

Study results will be available to the public on the mass.gov website. Results of the study will be disseminated through multiple channels: (1) peer reviewed journal publications; (2) presentations at national research meetings; (3) publicly available reports to the Center for Medicare and Medicaid Services; (4) publicly available summaries posted on the MassHealth website; and (5) directly to key stakeholders in ACO and CP leadership.

## Data Availability Statement

The raw data supporting the conclusions of this article will be made available by the authors, without undue reservation.

## Ethics Statement

The studies involving human participants were reviewed and approved by University of Massachusetts Medical School. Written informed consent for participation was not required for this study in accordance with the national legislation and the institutional requirements.

## Author Contributions

SG contributed to development of the protocol to study implementation of the DSRIP program and led writing of the manuscript. DG led development of the protocol, reviewed the manuscript, and approved the final version. MA, AK, and JN contributed to development of the protocol, revisions of the manuscript, and approved the final version of the manuscript. JH oversaw development of protocol, reviewed the manuscript, and approved the final version of the manuscript. All authors contributed to the article and approved the submitted version.

## Conflict of Interest

The authors declare that the research was conducted in the absence of any commercial or financial relationships that could be construed as a potential conflict of interest. The reviewer DR declared a shared affiliation, with no collaboration, with one of the authors, DG to the handling editor at the time of the Review.

## Abbreviations

ACO: accountable care organization
MA: Massachusetts
CP: Community Partner
LTSS: long term services and supports
DSRIP: Delivery System Reform Program
SWI: StateWide Investments
CFIR: Consolidated Framework for Implementation Research
MD: Medical Doctor
DO: Doctor of Osteopathy
NP: Nurse Practitioner
PA: Physician's Assistant
LPN: Licensed Practical Nurse
LICSW: Licensed Social Worker.
