# Supplementary material for: Barriers and Facilitators to Implementation of Value-Based Care Models in New Medicaid Accountable Care Organizations in Massachusetts: A Study Protocol
Source: Front Public Health. 2021 Apr 6;9:645665. doi: 10.3389/fpubh.2021.645665 (PMC8055830; doi:10.3389/fpubh.2021.645665)
Supplement: Supplementary file 1 [file Table_1.docx]

**Appendix A**

**Table A1: MassHealth ACO Models and Payment Arrangements**

| **ACO Type** | **n** | **Description** |
| --- | --- | --- |
| Accountable Care Partnership Plans (“Model A”) | 13 | - Partnership between a single health plan and provider-led ACO. - Receive monthly capitated payments from MassHealth based on enrollment and patient risk scores. - Take on full insurance risk for population. |
| Primary Care ACOs (Model B) | 3 | - Provider-led ACO contracts directly with MassHealth. - Use MassHealth’s network of providers. - Providers receive fee-for-services payments from MassHealth; ACOs accountable for performance but not insurance risk. |
| MCO-Administered ACO (Model C) | 1 | - Provider-led ACO contract with one or more of MassHealth’s MCOs. - MCO receives capitated payment from MassHealth; pays ACO according to MassHealth-approved arrangement. |
| Note: Table adapted from ACO program descriptions in the DSRIP protocol and 2018 DSRIP report. | | |

**Appendix B**

**Interview Guide - ACOs**

[Introduce Self] Thank you very much for taking the time to talk with me about your MassHealth ACO. As you know, we are part of the independent team that is evaluating the MassHealth ACO initiative. In this part of our study, we are trying to learn more about the approaches each ACO is taking to achieve the overall goals of the MassHealth ACO demonstration project: integrating and improving quality of care and reducing costs associated with caring for MassHealth beneficiaries. We know your healthcare organizations/ health plan may have more than one ACO contract, but we will be focusing only on your MassHealth ACO (insert name) in today’s discussion. This will be the first of two rounds of interviews with ACO senior leaders that we plan to conduct as part of the study – we anticipate administering the second round of interviews in about 2 years so that we can see how things have changed. Findings from these two rounds of interviews will be reported in the study interim and final reports respectively

Did you have a chance to review the fact sheet we sent ahead of time?

[if yes] Great. We want to remind you that we are recording the interviews to ensure that we accurately capture the information you provide. When we write our report about the interviews, we will not use the names, roles, or clinical practice site of respondents.

[If no, offer the fact sheet to interviewee and read script above without “As a reminder” lead]

Turn Recorder On

Do you have any questions before we start?

(Ice Breaker) First, I’d like to hear about your current role at (insert ACO name).

- What are the main responsibilities associated with your role or position
- How many years of healthcare experience in this and/or other settings

(Grand Tours)

Can you tell me a little about the factors that went into your organization’s decision to pursue a MassHealth ACO Contract?

What do you see as some of the most important changes your organization has needed to make in the process of implementing your ACO?

In general, how do you feel the MassHealth ACO initiative is working?

Are there patient populations that you feel have benefitted most in this first year?

Are there populations that you feel may have encountered greater challenges in the transition?

**SECTION 1: Organizational Structure**

1. What types of decisions are made at the MCO governance-level (in the case of Model A), ACO governance-level, or at the individual provider organization-level?

*Probe:*

What works well and less well about this division of decision-making

2. How has your ACO engaged MassHealth patients in governance?

*Probe:*

Role patients have in governance

What works well and less well

Adjustments made/planned for

3. How has your ACO engaged providers in governance?

*Probe*:

Roles providers have in governance

What works well and less well

Adjustments made/planned for

**SECTION 2: Provider Engagement**

**Some ACOs are using financial incentives for primary care providers, specialists, and/or hospitals as a mechanism to engage providers in delivery system changes.**

1. Is your ACO using financial incentives to engage providers in ACO-related changes?

2.If yes, please describe how your MassHealth ACO uses financial incentives to engage primary care providers in effective care delivery?

*Probe:*

Types of financial incentives (including alternative payment methods)

Types of providers exposed to risk

What works well/less well about these incentives

3. If yes, please describe how your ACO uses financial incentives to engage specialists and hospitals in effective care delivery?

*Probe:*

Types of financial incentives (including alternative payment methods)

Types of providers exposed to risk

What works well/less well about these incentives

4. If yes, please describe how are you are using other strategies, such as staff meetings or written communication strategies to engage providers and front-line workers in effective care delivery?

*Probe:*

Types of strategies

What has worked well/less well about these strategies

Provider knowledge of ACO care model in general

**SECTION 3: Care Coordination and Management**

1. We have reviewed several of the documents your ACO prepared for MassHealth, such as your participation plan, and learned about your ACO’s proposed strategies for coordinating care. How has your ACO implemented these strategies, specifically?

*Probe:*

Spread and uptake across ACO

Standardization of care coordination approach across provider orgs

2. Under what conditions are your ACO’s strategies for coordinating care working well and less well?

*Probe:*

How care coordination quality is assessed

3. What processes are in place to engage MassHealth patients in managing their own care?

*Probe:*

Degree to which these systems are common/vary across provider organizations:

4. Under what conditions are your systems for engaging MassHealth patients in care working well and less well?

*Probe:*

How patient engagement strategies are assessed

5. Excluding the CP assigned population for now (we ask separate questions about this population in a different portion of the interview), how does your ACO identify patients who would benefit from (or are eligible for?) case management?

*Probe:*

Case management strategies used

Standardization of identification and/or care management strategies across provider orgs.

6. Under what conditions are your strategies for managing (non-CP) high need/complex patients working well and less well?

*Probe:*

How care management effectiveness is assessed

7. (For Model A only): Can you tell us about your ACO’s progress in transitioning care management from (insert MCO name) to the ACO, specifically the estimated percent of patients whose care is now managed by your ACO?

8. In addition to working with CPs, what strategies are you using to coordinate and manage care for patients with BH needs?

*Probe:*

Successes, challenges

Access issues

Specific programs for patients with Opioid use disorders

9. How has your ACO approached screening for health-related social needs (HSRNs)?

*Probe:*

Screening tool used

Standardization of screening tool/approach across provider orgs.

Barriers/facilitators to HRSN screening

Issues about screening duplication/fatigue for patients

10. When Health Related Social Needs are identified, how does your ACO intervene?

*Probe:*

Referrals/services provided

Barriers/facilitators to addressing HRSNs

**SECTION 4: Quality and Process Improvement**

**The next set of questions are about quality and process improvement strategies, both for adult and pediatric Medicaid patients. First, I will ask about strategies related to adult patients, then ask if any of these strategies are different for pediatric patients.**

1. How would you characterize your ACO’s main strategies for managing/reducing care costs?

Degree to which strategies are common/differ across provider orgs.

Barriers and facilitators to managing/reducing care costs

Differences, if any, for pediatric patients

2. How would you characterize your ACO’s main strategies for meeting MassHealth’s ACO care quality performance metrics?

*Probe:*

Care processes

Health outcomes

Patient experience

Degree to which strategies are common/differ across provider orgs.

Barriers and facilitators to managing/reducing care costs

Differences, if any, for pediatric patients.

3. In what ways is your ACO’s health information technology supporting or inhibiting your ability to achieve performance goals?

*Probe:*

Ability to electronically share MassHealth patient info among ACO providers and with the MCO

Differences, if any, for pediatric patients

4. In general, how do you feel the transition to an ACO has impacted care to date?

*Probe:*

Conditions that impede/facilitate effective care under DSRIP/ACO model

Access to specialty care

Access for patients with complex needs

BH care

Differences, if any, for pediatric patients

5. Is there anything else you’d like to tell us about your ACO’s experience managing the performance metrics established under the ACO and DSRIP programs (e.g. quality measures) for adult or pediatric patients?

**SECTION 5: Community Partners**

**We know that ACOs may contract with multiple CPs, therefore if your experiences differ considerably between CP’s, please feel free to explain those differences in your answers**

1. Overall, how well is the CP program working for your ACO?

*Probe:*

Relative success of LTSS vs. BH CPs

Conditions that impede and facilitate CP program effectiveness

2. We understand that ACOs are expected to adopt systems for coordinating key administrative functions (e.g. enrollee identification and referrals, and enrollee outreach) with the MassHealth CPs they work with. Now that the program has launched, what do these systems look like?

*Probe*

Enrollee identification and referral systems

Enrollee outreach and engagement systems

Sharing information systems with MassHealth? With CPs?

3. How effective are the systems you describe for coordinating administrative functions?

*Probe*

Barriers and facilitators to coordinating administrative functions

4. We understand that ACOs are also expected to adopt systems for managing conflict resolution with the CPs they work with. Now that the program has launched, what do these systems look like?

5. How effective are the systems you describe for managing conflict resolution with CPs?

*Probe*

What works well/less well

6. We understand that ACOs are expected to adopt systems to coordinate “care coordination” with CPs to avoid duplicating care management and coordination efforts (i.e., following up with an enrollee after an avoidable hospital admission). Now that the program has launched, what do those systems look like?

7. How effective are the systems you describe for coordinating care?

*Probe*

Barriers and facilitators to coordinating care

**SECTION 6: Role of MassHealth and Environment**

1. How effective do you feel DSRIP funding has been in supporting care transformation at your ACO?

*Probe:*

Challenges accessing and spending DSRIP funding

Constraints on how DSRIP funds can be used

Need for additional financial or other resources

2. How is your ACO managing the intentional move towards sustainability, as opposed to reliance on DSRIP funds? (i.e. the decline over 5 years)

3. (If applicable) According to MassHealth data, we understand that your ACO has participated in the following Statewide Investments programs (*Insert programs*). What, if any, impact have these programs had on your ACO to date?

*Probe:*

Most beneficial/least beneficial about noted program(s)

4. Are there other ways MassHealth has supported (or impeded) your organization’s ability to operate as an ACO that we have not covered? Please explain.

*Probe:*

Written and other types of guidance

Reporting burden

Data supports

5. How have other local, state, or federal policies -- that is, apart from MassHealth ACO policies -- helped or hindered your organization’s ability to operate effectively as a MassHealth ACO?

*Probe:*

Other payer reporting requirements that align/conflict

Other funding/initiatives that align/conflict (e.g., Health Policy Commission’s SHIFT-Care grant program)

Privacy/information sharing policies

**Thank you for your time and insights.**

**Is there anything else you think is important for our team to know about your experience with the MassHealth ACO Demonstration Project and/or experience operating as a MassHealth ACO?**

**Interview Guide - CPs**

[Introduce Self] Thank you very much for taking the time to talk with us about your organization’s participation in MassHealth’s Community Partner (CP) program, which is closely tied the Medicaid ACO initiative. As you know, we are part of the independent team that is evaluating and assessing the MassHealth Delivery System Reform Incentive Program (DSRIP) program that includes evaluating both the ACO and CP programs. In this part of our study, we are trying to learn more about the approaches CPs are taking to achieve the overall goals of the DSRIP program, integrating and improving quality of care and reducing costs for Medicaid patients. This will be the first of two rounds of interviews with CP senior administrators that we plan to conduct as part of the study – we anticipate administering the second round of interviews in about 2 years so that we can see how things have changed. Findings from these two rounds of interviews will be reported in the study mid-point and final reports respectively.

Did you have a chance to review the fact sheet we sent ahead of time?

[If yes] Great. We want to remind you that we are recording the interviews to ensure that we accurately capture the information you provide. When we write our report about the interviews, we will not use the names, roles, or clinical practice site of respondents.

[If no, offer the information sheet to interviewee and read script above without “As a reminder” lead]

Turn Recorder On

Do you have any questions before we start?

(Ice Breaker) First, I’d like to hear about your role in your organization.

- What are the main responsibilities associated with your role or position
- How many years of healthcare experience in this and/or other settings

(Grand Tours)

What do you see as some of the most important changes your organization has needed to make to operate as a MassHealth CP?

In general, how well do you feel the MassHealth CP program is working?

- And for what types of patients is the program working especially well; and for what types is it working less well?

We know that CPs contract with multiple ACOs, therefore if your experiences differ considerably between ACOs, please feel free to explain those differences in your answers throughout the interview. [Return to this as probe throughout interview]

**SECTION 1: Alignment with ACO(s) and other Consortium/Affiliated CPs**

1. We understand that CPs, through their Documented Processes, are expected to adopt systems for coordinating administrative functions with the ACOs they work with, including enrollee assignment, patient outreach and engagement, and exchanging information about shared patients. Now that the program has launched, how well are these systems functioning?

*Probe:*

Enrollee assignment systems

Patient outreach and engagement systems

Sharing information systems

Differences from original plan

2. Under what conditions are your systems for coordinating administrative functions with the ACOs you work with working well and less well?

*Probe:*

Provide examples where things worked well/not well

3. We understand that CPs are expected to adopt systems to minimize duplicating care management/coordination efforts with the ACOs they work with (for instance, following up with a patient after an avoidable hospital admission). How well are these systems functioning?

*Probe:*

Coordinating care coordination systems

Differences from original plan

Barriers and facilitators to coordinating care coordination efforts

4. We understand that CPs are also expected to include plans for managing conflict with the ACOs they work with in their Documented Processes. Have you needed to implement these plans, and if so, how are they working?

*Probe:*

Conflict resolution systems

Differences from original plan

Barriers and facilitators to managing conflict

5. Does your organization work with Adult Community Clinical Service (ACCS) enrollees?

6. If yes, do any of the systems that we have been discussing differ for your interactions with providers that work with your ACCS enrollees?

*Probe:*

If yes, please explain

7. (If applicable): We understand that your organization is part of a consortium/affiliation of CPs. What strategies does your organization use to engage consortium/affiliated partners in delivering care coordination supports to your enrollees?

*Probe:*

Barriers and facilitators to constituent engagement

**SECTION 2: Care Management and Transitions**

1. We understand that CPs are required to develop a care plan for their enrollees. For your ACO enrollees, can you tell us the systems you have in place for doing that?

*Probe*

Staff involved

Coordination with ACO

How well the process is working

2. How does your organization help MassHealth patients navigate the BH/LTSS delivery system?

*Probe:*

Barriers and facilitators to effective patient navigation

How effectiveness of patient navigation practices is assessed

3. What strategies does your organization use to promote meaningful enrollee participation and engagement with their care?

*Probe:*

Barriers and facilitators to patient engagement

How utility of patient engagement strategies is assessed

4. What systems have you adopted to coordinate care across the clinical and social service providers that serve MassHealth patients with complex BH/LTSS needs?

*Probe:*

Barriers and facilitators to care coordination

Use of electronic data transfer

5. We understand that CPs are expected to assist their MassHealth partner ACOs/MCOs to better leverage existing BH/LTSS community resources in caring for patients with BH/LTSS needs. Can you comment on any activities in this area to date?

*Probe:*

Ask for examples

**SECTION 3: Workforce Development**

1. We are interested in understanding how your organization went about recruiting, training, and retaining staff for the new ACO/MCO partnerships. Let’s start with recruitment. Please explain.

*Probe:*

Training

Retaining

2. What has gone well and less well about your efforts to recruit, train and retain staff?

*Probe:*

Barriers and facilitators to recruiting staff

Barriers and facilitators to training staff

Barriers and facilitators to retaining staff

**SECTION 4: Quality and Process Improvement**

1. What strategies has your organization adopted to meet the CP quality performance benchmarks under the MassHealth CP program?

*Probe:*

Barriers and facilitators to meeting performance metrics

Barriers and facilitators to aligning efforts

2. What strategies has your organization adopted to engage both leadership and front-line staff in your CP in working to meet CP performance goals?

*Probe:*

Financial incentives

Other types of incentives (such as quality improvement feedback loops)

Barriers and facilitators to staff engagement

**SECTION 5: Role of MassHealth and Environment**

1. To what degree has DSRIP funding allowed your organization to create the infrastructure needed to operate as a CP?

*Probe:*

Challenges accessing and spending DSRIP funding

Need for and availability of additional financial resources

2. (If applicable): We understand from MassHealth that your CP participated in the following State-Wide Investment programs (insert name of programs that CP has participated in). What if any impact has this (or these) program(s) had on your CP?

*Probe:*

Most beneficial/least beneficial about noted program(s)

3. Are there other ways MassHealth has supported (or impeded) your organization’s ability to operate as a CP? Please explain.

Written or other types of guidance

Reporting burden

Data supports

Any suggested modifications for program improvement

4. How have other local, state, or federal policies helped or hindered your organization’s ability to operate effectively as a MassHealth CP (for example, is your CP participating in any other state-funded programs that are aligned or in conflict with the MassHealth CP initiative)?

*Probe:*

Other payer reporting requirements that align/conflict

Other funding/Initiatives that align/conflict

**Thank you for your time and insight.**

**Is there anything else you think is important for our team to know about your experiences as a CP in the new MassHealth ACO program?**

**Interview Guide - MassHealth Patients - Adult**

[Introduce Self]

Thank you so much for agreeing to talk to me today about your experience as a MassHealth Patient enrolled in XXXXXX (ACO organization, if known). XXXXX is one of the 17 MassHealth Accountable Care Organizations, or ACOs, which were created about two years ago in the hopes of improving the way your healthcare is delivered. I’m part of the team that is evaluating the MassHealth ACO program, and we are going to be looking at many aspects of the ACO program over the next few years. In this part of our project, we want to learn more about patient experiences with the health system (MassHealth). What we learn from these interviews will be reported in our two study reports.

Did you have a chance to review the fact sheet we sent ahead of time?

[If yes] Great. We want to remind you that we are recording the interviews to ensure that we accurately capture the information you provide. When we write our report about the interviews, we will not use personal information like names, addresses, dates or month of medical care, or birthdays.

[If no, offer the fact sheet to interviewee and read script above without “As a reminder” lead]

*If you have any questions about the Coronavirus pandemic/COVID-19, please use the following:*

- **Patients with COVID-19 related health questions should be directed to call their provider's office or 2-1-1 for guidance.**
- General eligibility and enrollment questions should go to **MassHealth CSC**:
  - Call: ([800) 841-290](tel:+18008412900)0
  - TTY: (800) 497-4648
  - Hours: Monday - Friday, 8am till 5pm
- ​In general, managed care patients with access to care issues can contact **My Ombudsman**:
  - ​Call: (855) 781-9898; VideoPhone: (339) 224-6831; TTY: use MassRelay at 711
  - Hours: Monday–Friday, 9 a.m.–4 p.m.
  - Email: [info@myombudsman.org](mailto:info@myombudsman.org)
  - Or visit: [http://www.myombudsman.org](https://nam01.safelinks.protection.outlook.com/?url=http%3A%2F%2Fwww.myombudsman.org%2F&data=02%7C01%7CAparna.Kachoria%40umassmed.edu%7C61f8d42be6694d3e44f508d7d0068ab9%7Cee9155fe2da34378a6c44405faf57b2e%7C0%7C0%7C637206598825681017&sdata=31snEXaPwtMLVBfRKnEC1jIZeqO0lGENc8y8lQOr7zE%3D&reserved=0)

**[Note to Interviewer: Please remind interviewee that they should not provide any identifying details such as dates of care received, names of doctors, or other identifying information.****If an interviewee gives such details, you will need to stop the recording, rewind the recorder to the beginning of the question, and re-ask the question while reminding the interviewee not to provide such details.]**

**Turn Recorder On**

Do you have any questions before we start?

(Ice Breaker) First, I’d like to learn a little bit about you and your healthcare needs.

*Probes*: Health concerns, other concerns, what kinds of providers do they see (PCP, specialists etc.)

- How would you describe your health?
- Where do you go for routine care? Do you have a primary care provider? Who are they? Have you seen them for more than a year, and if so, how many years have you been seeing them? Without identifying any dates or specific months, how often do you see them?
- Has your health changed over the last 3 years?
- You mentioned on your nomination form that you have X (condition)? How long have you had that? Please do not tell us any specific dates on which you received care or a diagnosis.

How long have you been on MassHealth?

- BH Only: You had mentioned in your nomination form and screening call that you have behavioral health needs [mention reference to diagnoses or symptoms]. Do you have any supports to help you manage those needs?
  - Probes: Tell me about the supports you have. Assess for formal vs. informal supports
  - Do you have any community agency or case manager involved in helping you with you needs?
  - If you need to access behavioral health supports in the community, what has been easier and/or more challenging about getting the services you need?
- Do you have a case manager or coordinator? [pause] This is an individual or team of people who are involved in coordinating or helping you with your care.
  - *Probes:* Tell me a bit about this person or people and what they do for you or help you with.
  - Are there times when they ask or do the same things?
  - Where do you meet with your care coordinator? Do they come to your home?
- Do you receive any services at home? Please do not tell us any specific dates or months in which you received services at home.
  - *Probes:* How is your experience with your in-home services?
    - What types of services? PCA?

***Enrollment (Workgroup as well as EDD re: Patient Engagement)***

**The MassHealth ACO program started about two years ago. Our first questions are about the process you experienced when you enrolled in XXXXXX, your ACO (if known, use organization name). [Note to Interviewer: Please remind interviewee that they should not provide any identifying details such as dates or month of care received, names of doctors, or other identifying information.]**

1. Let’s talk about ACOs. Before today, did you know that you were enrolled in an ACO? [pause] From your perspective, what does it mean to belong to an ACO?

*Probes*:

- What is the role of the ACO?
- How does your ACO fill that role?

1. I’d like to ask you about the process of getting started with your ACO [If the Patient is unsure what ACO organization they are in, tell them that is ok and move on to some of the probes]

Did Patient know what ACO Organization they an enrolled in? Yes No

*Probes*:

- What were the easy parts about getting started? What about the difficult parts? (paperwork, stops in services/delays, other issues)
- What was the process to get your primary healthcare provider?
- Did you keep the same primary heath care provider you had before?

1. [If in CP] Let’s talk for a few minutes about Community Partners. Based on your experience, how would you define or describe a Community Partner? [If known, reference CP by name]

*Probes*:

- What is the role of the CP? How does your CP provide supports to fill that role?
- Can you talk about how you started working with your care coordinator at your CP? How long have you been working with them?
- Did you experience any barriers to engaging with your CP? What was easy about starting services with them? What was difficult?

***Care Experience (Workgroup and EDD re: Patient Experience, Care Coordination/ Integration)***

**One of the goals of the MassHealth ACO was to create a better care experience for Patients. This included things like more communication between providers to make sure you are getting what you need. Providers are the people who are involved in your care, and can include doctors, nurses and social workers or case managers. [Note to Interviewer: Please remind interviewee that they should not provide any identifying details such as dates or month of care received, names of doctors, or other identifying information.]**

1. How do you feel about your healthcare in general?
2. In your opinion, what is a good or a bad healthcare experience? Alternate wording: What does good or bad care mean to you?

1. Can you tell me about what a typical visit to your doctor or primary care provider is like? (This is the doctor that you see most often, and could be a primary care provider, specialist, or other type of medical professional). Please do not identify any specific dates or months you saw them.

*Probes:*

- How recently was your last visit to your primary care provider [or doctor you see most often]?
- Would you say that was a “typical” visit?
- What do you talk about? How comfortable are you about asking them questions?
- What questions do they ask you? What questions do you ask them?
- What services do they help you with?
- How would you describe your experience with your provider overall?
- Do you have suggestions for improvements?
- Was this a good or a bad healthcare experience?

1. You mentioned earlier that you are seeing XXXXXX provider for your XXXXX (ex: a cardiac specialist for your heart condition and blood pressure).

*Probes:*

- How recently was your last visit to this provider? Please do not identify the date or month.
- Thinking about your last visit to the provider for X, Why were you there? Would you say that was a “typical” visit?
- What do you talk about? What questions do they ask you? What questions do you ask them? How comfortable are you about asking them questions?
- What services do they help you with?
- How would you describe your experience with your provider overall?
- Do you have suggestions for improvements?
- Was this a good or a bad healthcare experience?

Another part of the MassHealth ACO program was to make sure Patient’s care was coordinated, meaning that your providers and staff who work with you were talking to each other to identify and figure out how to meet your needs.

1. Do you think that your other providers know information you have told your PCP, or do you have to share information over and over again?

*Probes:*

- What questions or information do you feel you have to share over and over? How does this make you feel?
- What about tests/assessments?
- What types of information are they sharing (right information right time)? How helpful is this?

1. Many people get their care by going into a doctor’s office or clinic, but it’s possible to get care other ways like through the phone or internet. How do you get care (face to face vs other means)? [pause]
2. How does your doctor work with you to get you the care you want or need?

*Probes:*

- Do you feel your doctor understands your needs? How do you make sure your doctor understands your needs, both medical and not medical (like housing, transportation, nutrition)?
- How do you work with your providers to explain your needs?
- Who helps you with things like housing or transportation?
- Who helps you decide what care you should get?
- How easy or difficult is it for you to get the authorizations for other services you need, like personal care, nursing or psychiatric care?
- *Must Probe: Who is on your care team?*

1. I want to hear a little more about how you set goals for your care, and how you make decisions about your health and your treatment. You mentioned just now that you consider XXXXXXXX as patients of your care team. Can you explain what conversations you have with your care team to determine your healthcare goals?

*Probes:*

- How do you decide what is important?
- What are your goals?
- How is your care team working with you to meet your goals? How do they help you make decisions about your health?
- What gets in the way of meeting your goals?

1. Sometimes it can be confusing to understand what care you can receive through MassHealth. Can you tell me how your care team helps you understand your care?

*Probes:*

- What kind of information do they provide you?
- Do they talk about how what you tell them might be shared with other individuals (doctors, therapist, nurses, care coordinator, etc.)? What do they tell you?
- What type of information is really helpful for you? What type of information is not helpful for you?
- How would you like to receive information?
- What type of information would you like to receive?
- [If they say no to above] Where/how else are you finding information if it is not being shared with you?

1. In what year was the last time you went to the hospital, either planned or unplanned?

*Probes:*

- Thinking about your most recent trip to the hospital, what was the discharge process like?
- When you were discharged from the hospital, who contacted you right afterwards?
- What services were you offered? What services did you choose/receive?

1. What was the transition like for you when you left the hospital and went back home?

*Probes*:

- Were services set up for you to support your return home such as home healthcare, nursing or physical therapy? Who set up these services for you?
- Any unmet needs? Lingering health concerns?
- Were you contacted by too many people? Not enough/the right people?

1. We mentioned earlier that the MassHealth ACO program started about two years ago. That means before, you might not have been in an ACO. In what ways, if any, is your care different now than before? Say, two or three years ago?

*Probes:*

- In what ways is it different?
- How is it the same?
- What is better or worse, and why?

***Health Status (EDD and workgroup)***

**In the next couple of questions I’m interested in learning about your feelings about your medical/physical health. [Note to Interviewer: Please remind interviewee that they should not provide any identifying details such as dates or month of care received, names of doctors, or other identifying information.]**

1. How is your health overall? What else do you think impacts your health?
   *Probes:*

- You mentioned earlier that your health [has changed, stayed consistent, etc.] over the past few years. You said [something happened, nothing happened]. Since [something happened—do not identify dates/months], how often do you go to the doctor/ER before that [thing happened, your health changed]
- Other issues besides ACO/CP [ex: HRSNs, flex services, finances]

1. [CP Only] You told me about your experience in working with XXXXX, your CP. For the next few questions, I’d like to learn about how your Mental/Behavioral Health services, and/or the ways your LTSS supports impact your health. Can you tell me how your health is overall now that you are using CP services?

*Probes:*

- What else do you think impacts your health?
- Has it changed? How?

1. Overall, how has your quality of life changed in the last year because of your health status or healthcare?

- Probe for improvements (or not) in social situation, services, HRSNs

***Community Partners (EDD)***

**As part of the MassHealth ACO program, ACOs have relationships with community-based organizations called Community Partners. These partnerships are another part of the goal of meeting patients’ needs and coordinating their care between providers, whether it’s for medical care, mental/behavioral health, or long-term services and supports to help them stay in the community such as a personal care attendant or a visiting nurse. When we first started talking, you mentioned that you have XXXXXX [reference if they noted a mental health diagnosis and/or disability that requires LTSS support]. To help you manage those needs, you are getting supports from XXXXX [note CP name if known]. [Note to Interviewer: Please remind interviewee that they should not provide any identifying details such as dates or month of care received, names of doctors, or other identifying information.]**

1. Do you know what CP you receive services from?

Probes: How did you hear about your CP?

What do you repatient about the enrollment process with your CP?

Where did you first meet your CP staff?

1. Tell me about what getting services from your CP typically looks like.

*Probes:*

- What services do they help you with?
- What do you talk about?
- What questions do they ask you? What questions do you ask them?
- How do you make decisions about your care plan?

1. How does your CP help you with getting other services?

*Probes:*

- Does your CP help you with other needs (like housing, transportation, food)?
- Who else helps you with those needs?

1. We asked you earlier about how you make your needs known to your doctors and medical providers, and how those providers help you to understand your care. In thinking about your CP can you also tell me how the CP staff helps you to understand services available to help you with your care and/or needs?

*Probes:*

- What kind of information do they give you?
- What kind of information is really helpful? What is not?
- How would you like to receive information?
- What type of information would you like to receive that you haven’t gotten?

1. We also talked about how your providers help you in setting and meeting your care goals. How does the CP help you make decisions about your goals?
2. Are there any goals that you have that you think both your PCP and the CP can help you with?

- How does your CP work with your PCP or ACO?

1. The goals you set with your providers are usually written down as part of your plan of care. Do you know what a care plan is?

*Probes:*

- What is in your care plan?
- Do you have a copy of your care plan?

***Care Integration/Info Sharing (EDD)***

**Another goal of the MassHealth ACO was to better integrate care for Patients. Integration means bringing people or things together.**

1. Until two years ago or maybe even less, you might not have been in an ACO. When [Primary care doctor] refers you to see another provider now, what, if anything, is different in how those providers share and understand your information?
2. Can you talk about your experiences with getting the care/services you want or need when you need them?

*Probes:*

- Do you experience any delays in getting care?
- How do you work with different patients of your care team(providers) to get the care you want?
- What is working well? Not so well?
- What care are you not getting that you want/need?
- Why do you think you aren’t receiving them? (probe for availability/accessibility disability, language ,)
- Can you talk about getting care when you are sick now that you are in an ACO (is it different than two or three years ago? Changes?)
- Can you talk about getting care when you are healthy now that you are in an ACO? (Is it different than before? Changes?)

**COVID-19 Considerations***

We know that the novel coronavirus and the current epidemic of COVID-19 may have contributed to changes in the healthcare system.

1. Have you noticed any changes to your care over the last few weeks?

2. Has anyone been in touch with you about your care (in the context of COVID-19)? If so, how? What information? [e.g., telehealth?] How to get in contact with your provider?

3. Are you continuing to see your provider for routine visits? (think medically complex folks who may have frequent visits routinely) [Any trouble seeing your provider?]

4. Oh, you’re not going into the office. Are there other ways you are in touch with your PCP, such as telehealth options including telephone or videoconferencing visits?

- Have you used telehealth (i.e. seen the doctor over the phone/video calling)?
  - Are you using technology such as FaceTime or another smartphone app? Or a service like Zoom or Skype?
- Were you given the option to see your doctor/provider in person or having a telehealth appointment or was this choice made for you?
- How many telehealth visits have you had over the last couple weeks/month?
  - Do these meet your definition of a “good care visit”? Are you satisfied with them?
  - If you needed to, were you able to go to the office/hospital (maybe for lab tests or vaccinations)?
- Which other providers would you want to see via telehealth?
- Do you think you would want to make telehealth part of your regular care routine after Covid-19?
- Have you had any issues with telehealth, for example with internet access or having the right equipment like a smartphone? Did you prefer one method over another?

5. Can you continue to focus on your health issues (or are you "distracted" by COVID-19)?

[Information from MassHealth – reference again for their awareness]

- General eligibility and enrollment questions should go to **MassHealth CSC**:
  - Call: ([800) 841-290](tel:+18008412900)0
  - TTY: (800) 497-4648
  - Hours: Monday - Friday, 8am till 5pm
- ​Patients with COVID-19 related health questions should be directed to call their provider's office or 2-1-1 for guidance.
- In general, managed care patients with access to care issues can contact **My Ombudsman**:
  - ​Call: (855) 781-9898; VideoPhone: (339) 224-6831; TTY: use MassRelay at 711
  - Hours: Monday–Friday, 9 a.m.–4 p.m.
  - Email: [info@myombudsman.org](mailto:info@myombudsman.org)
  - Or visit: [http://www.myombudsman.org](https://nam01.safelinks.protection.outlook.com/?url=http%3A%2F%2Fwww.myombudsman.org%2F&data=02%7C01%7CAparna.Kachoria%40umassmed.edu%7C61f8d42be6694d3e44f508d7d0068ab9%7Cee9155fe2da34378a6c44405faf57b2e%7C0%7C0%7C637206598825681017&sdata=31snEXaPwtMLVBfRKnEC1jIZeqO0lGENc8y8lQOr7zE%3D&reserved=0)

Is there anything else that you would like to share with us today regarding your experiences as a MassHealth ACO patient?**[Note to Interviewer: Please remind interviewee that they should not provide any identifying details such as dates or month of care received, names of doctors, or other identifying information.]**

Thank you for your time today.

**[Turn Recorder Off]**

To better understand patient experience, we are asking some **optional** demographic information questions. You do not have to answer any question you do not want to.

[Go through demographic form questions]

As a token of our appreciation, we will be sending you your gift card via mail or email. Which would you prefer?

[Note down email address or physical address]

Thank you again for your time. We really appreciate your perspective and input.

*** COVID-19 Questions Were Added April 2020**

**Interview Guide - MassHealth Patients – Pediatric**

[Introduce Self]

Thank you so much for agreeing to talk to me today about your child’s experience as a MassHealth Patient enrolled in XXXXXX (ACO organization, if known). XXXXX is one of the 17 MassHealth Accountable Care Organizations, or ACOs, which were created about two years ago in the hopes of improving the way healthcare is delivered for your child. I’m part of the team that is evaluating the MassHealth ACO program, and we are going to be looking at many aspects of the ACO program over the next few years. In this part of our project, we want to learn more about patient experiences with the health system (MassHealth). What we learn from these interviews will be reported in our two study reports.

Did you have a chance to review the fact sheet we sent ahead of time?

[If yes] Great. We want to remind you that we are recording the interviews to ensure that we accurately capture the information you provide. When we write our report about the interviews, we will not use personal information like names or birthdays.

[If no, offer the fact sheet to interviewee and read script above without “As a reminder” lead]

**[Note to Interviewer: Please remind interviewee that they should not provide any identifying details such as dates or month of care received, names of doctors, or other identifying information. If an interviewee gives such details, you will need to stop the recording, rewind the recorder to the beginning of the question, and re-ask the question while reminding the interviewee not to provide such details.]**

**Turn Recorder On**

Do you have any questions before we start?

(Ice Breaker) First, I’d like to learn a little bit about your child and her/his healthcare needs.

*Probes*: Health concerns, other concerns, what kinds of providers do they see (PCP, specialists etc.)

- How would you describe your child’s health?
- Where does your child go for routine care? Does your child have a primary care provider? Who is it? Has your child been seeing them for more than a year, and if so, how many years? Without identifying any dates or specific months, how often do you see them?
- In what ways, if any, has your child’s health changed over the last 3 years?
- You mentioned on your nomination form that your child has X (condition). How long has your child had that? Please do not tell us any specific dates on which your child received care or a diagnosis.
- How long has your child been insured by MassHealth?

Does your child have a case manager or care coordinator? This is an individual or team of people who are involved in coordinating or helping you with your child’s care.

*Probes:* Tell me a bit about this person or people and what they do for your child or help you/your child with.

Are there times when they ask or do things over and over again?

Where do you meet with your care coordinator? Do they come to your home?

What services, if any, does your child receive at home? Please do not tell us any specific dates or months in which your child received services at home.

*Probes:* How is your experience with your in-home services?

***Enrollment (Workgroup as well as EDD re: Patient Engagement)***

**The MassHealth ACO program started about two years ago. Our first questions are about the process you experienced when your child was enrolled in XXXXXX, your ACO (if known, use organization name). [Note to Interviewer: Please remind interviewee that they should not provide any identifying details such as dates or month of care received, names of doctors, or other identifying information.]**

1. Let’s talk about ACOs. From your perspective, what does it mean to belong to an ACO?

*Probes*:

- What is the role of the ACO?
- How does your ACO fill that role?

1. I’d like to ask you about the process of getting started with your ACO [If the Patient is unsure what ACO organization they are in, tell them that is ok and move on to the probes]

Did Patient know what ACO Organization they an enrolled in? Yes No

*Probes*:

- What were the easy parts about getting started? What about the difficult parts? (paperwork, stops in services/delays, other issues)
- What was the process to get your child’s primary healthcare provider?
- Did your child keep the same primary healthcare provider they had before joining the ACO?

1. [If in CP] Let’s talk for a few minutes about Community Partners. Based on your experience, how would you define or describe a Community Partner? [If known, reference CP by name]

*Probes*:

- What is the role of the CP in your child’s healthcare? How does your CP provide supports to fill that role? Do you feel that the CP is providing helpful services specific to your child’s needs?
- Can you talk about how you started working with your care coordinator at your CP? How long have you been working with them?
- Did you experience any barriers to engaging with your CP? What was easy about starting services with them? What was difficult?

***Care Experience (Workgroup and EDD re: Patient Experience, Care Coordination/ Integration)***

**One of the goals of the MassHealth ACO was to create a better care experience for Patients. This included things like more communication between providers to make sure you are getting what you need. Providers are the people who are involved in your care, and can include doctors, nurses and social workers or case managers. [Note to Interviewer: Please remind interviewee that they should not provide any identifying details such as dates or month of care received, names of doctors, or other identifying information.]**

1. How do you feel about your child’s healthcare in general?
2. In your opinion, what is a good or a bad healthcare experience? Alternate wording: What does good or bad care mean to you?

1. Can you tell me about what a typical visit to your child’s doctor or primary care provider is like? (This is the doctor that your child sees most often, and could be a primary care provider, specialist, or other type of medical professional) Please do not identify any specific dates or months you saw them.

*Probes:*

- How recently was your last visit to your child’s primary care provider [or doctor your child sees most often] and what was the reason for the visit?
- Would you say that was a “typical” visit?
- What do you talk about? How comfortable are you about asking them questions? [If pediatric patient is 10 years or older: How comfortable is your child asking questions?]
- What questions do they ask you? What questions do you ask them?
- What services do they help you with?
- Was this a good or a bad healthcare experience?
- How would you describe your experience with your provider overall?
- Do you have suggestions for improvements?

1. You mentioned earlier that you are seeing XXXXXX provider for your child’s XXXXX (ex: a cardiac specialist for your heart condition and blood pressure).

*Probes:*

- How recently was your last visit to this provider? Please do not identify the date or month.
- Thinking about your last visit to the provider for XXXX, why were you there? Would you say that was a “typical” visit?
- What do you talk about? What questions do they ask you? What questions do you ask them? How comfortable are you/your child about asking them questions?
- What services do they help you with?
- How would you describe your experience with your provider overall?
- Do you have suggestions for improvements?
- Was this a good or a bad healthcare experience?

Another part of the MassHealth ACO program is to make sure every patients’ care is coordinated, meaning that the providers and staff who work with you are talking to each other to identify and figure out how to meet your needs.

1. Do you think that your child’s other providers know information you have told your child’s PCP, or do you have to share information over and over again?

*Probes:*

- What questions or information do you feel you have to share over and over? How does this make you feel?
- Does your child have to repeat tests/assessments?
- What types of information are they sharing (right information right time)? How helpful is this?

1. Many people get their care by going into a doctor’s office or clinic, but it’s possible to get care other ways like through the phone or internet. How do you get care (face to face vs other means) for your child? [pause]
2. How does your doctor work with you/your child to get you the care you want or need?

*Probes:*

- Do you feel your doctor understands your/your child’s needs? How do you make sure your doctor understands your/your child’s needs, both medical and not medical (like housing, transportation, nutrition)?
- How do you work with your providers to explain your/your child’s needs?
- Who helps you with things like housing or transportation?
- Who helps you decide what care you/your child should get?
- How easy or difficult is it for you to get the authorizations for other services you/your child needs, like personal care, nursing or psychiatric care?
- *Must Probe: Who is on your child’s care team?*

1. I want to hear a little more about how you set goals for your child’s care, and how you make decisions about your child’s health and your treatment. You mentioned that you consider XXXXXXXX as patients of your child’s care team. Can you explain what conversations you have with the care team to determine your child’s healthcare goals?

*Probes:*

- How do you decide what is important? What influences these decisions?
- What are your goals?
- How is your child’s care team working with you to meet your goals? How do they help you make decisions about your child’s health?
- What gets in the way of meeting your goals?

1. Sometimes it can be confusing to understand what care your child can receive through MassHealth. Can you tell me how your child’s care team helps you understand your child’s care?

*Probes:*

- What kind of information do they provide you?
- Do they talk about how what you tell them might be shared with other individuals (doctors, therapist, nurses, care coordinator, etc.)? What do they tell you?
- What type of information is really helpful for you? What type of information is not helpful for you?
- How would you like to receive information?
- What type of information would you like to receive?
- [If they say no to above] Where/how else are you finding information if it is not being shared with you?

1. Was the last time your child went to the hospital, either planned or unplanned?

*Probes:*

- Thinking about your most recent trip to the hospital, what was the discharge process like for your child?
- When your child was discharged from the hospital, who, if anyone, contacted you right afterwards?
- [If they were contacted] What services were you/your child offered? What services did you/your child choose/receive?
- [If they were not contacted] Would you have liked to have been contacted? Did you know you were supposed to be contacted? What information would you have liked to have received?

1. What was the transition like for your child when they left the hospital and went back home?

*Probes*:

- Were services were set up for you to support your child’s return home? These could be things like home healthcare, nursing or physical therapy. Who set up these services for you?
- Does your child have any unmet needs? Does your child have any lingering health concerns?
- Were you contacted by too many people? Not enough/the right people?

1. We mentioned earlier that the MassHealth ACO program started about two years ago. That means before then, you might not have been in an ACO. In what ways, if any, is your child’s care different now than before? Say, two or three years ago?

*Probes:*

- In what ways is it different?
- How is it the same?
- What is better or worse, and why?

***Health Status (EDD and workgroup)***

**In the next couple of questions I’m interested in learning about your feelings about your child’s medical/physical health. [Note to Interviewer: Please remind interviewee that they should not provide any identifying details such as dates or month of care received, names of doctors, or other identifying information.]**

1. How is your child’s health overall? What else do you think impacts your child’s health?

*Probes:*

- You mentioned earlier that your child’s health [has changed, stayed consistent, etc.] over the past few years. You said [something happened, nothing happened]. Since [something happened—do not identify dates/months], how often do you go to the doctor/ER before that [thing happened, your health changed]
- Do you have any other issues besides ACO/CP [ex: HRSNs, flex services, finances, school/education issues] that you would like to discuss in relation to your child’s health?

1. [CP Only] You told me about your experience in working with XXXXX, your CP. For the next few questions, I’d like to learn about your feelings about your child’s Mental/Behavioral Health services, and/or the ways your LTSS supports impact your child’s health. Can you tell me how your child’s health is overall now that you are using CP services?

*Probes:*

- What else do you think impacts your health?
- Has it changed? How?

1. Overall, how has your child’s quality of life changed in the last year because of your child’s health status or healthcare?

- Probe for improvements (or not) in social situation, services, HRSNs
- Do you feel anything can be done to better your child’s health and quality of life?

***Community Partners (EDD)***

**As part of the MassHealth ACO program, ACOs have relationships with community-based organizations called Community Partners. These partnerships are another part of the goal of meeting patients’ needs and coordinating their care between providers, whether it’s for medical care, mental/behavioral health, or long-term services and supports to help them stay in the community. When we first started talking, you mentioned that you have XXXXXX [reference if they noted a mental health diagnosis and/or disability that requires LTSS support]. To help you manage those needs, you are getting supports from XXXXX [note CP name if known]. [Note to Interviewer: Please remind interviewee that they should not provide any identifying details such as dates or month of care received, names of doctors, or other identifying information.]**

1. Do you know what CP your child receive services from?

Probes: How did you hear about your CP?

What do you repatient about the enrollment process with your CP?

Where did you first meet your CP representative/staff patient?

1. Tell me about what getting services from your CP for your child typically looks like.

*Probes:*

- What services do they help you with?
- What do you talk about?
- What questions do they ask you? What questions do you ask them?
- How do you make decisions about your child’s ?

1. How does your CP help your child with getting other services?

*Probes:*

- Does your CP help you with other needs (like housing, transportation, food)?
- Who else helps you with those needs?

1. We asked you earlier about how you make your child’s needs known to your doctors and medical providers, and how those providers help you to understand your care. In thinking about your CP can you also tell me how the CP staff helps you to understand services available to help you with your child’s care and/or needs?

*Probes:*

- What kind of information do they give you?
- What kind of information is really helpful? What is not?
- How would you like to receive information?
- What type of information would you like to receive that you haven’t gotten?

1. We also talked about how your providers help you/your child in setting and meeting your care goals. How does the CP help you/your child make decisions about your goals?
2. Are there any goals that you have that you think both your PCP and the CP can help you or your child with?

- How does your child’s CP work with your child’s PCP or ACO?

1. The goals you set with your child’s providers are usually written down as part of your child’s plan of care. Do you know what a care plan is?

*Probes:*

- What is in your child ‘s care plan?
- Do you have a copy of your child’s care plan?

***Care Integration/Info Sharing (EDD)***

**Another goal of the MassHealth ACO was to better integrate care for Patients. Integration means bringing people or things together.**

1. Until two years ago or maybe even less, your child might not have been in an ACO. When [Primary care doctor] refers you to see another provider now, what, if anything, is different in how those providers share and understand your child’s information?
2. Can you talk about your experiences with getting the care/services your child wants or needs when you need them?

*Probes:*

- Do you experience any delays in getting care for your child?
- How do you work with different patients of your care team(providers) to get the care you want for your child?
- What is working well? Not so well?
- What care are you not getting for your child that you want/need?
- Why do you think you aren’t receiving them? (probe for availability/accessibility disability, language)
- Can you talk about the process for getting care when your child is sick now that you are in an ACO (is it different than two or three years ago? Changes?)
- Can you talk about getting care when your child is healthy now that you are in an ACO? (Is it different than before? Changes?)

***COVID-19 Considerations****

We know that the novel coronavirus and the current epidemic of COVID-19 may have contributed to changes in the healthcare system.

1. Have you noticed any changes to your care over the last few weeks?

2. Has anyone been in touch with you about your care (in the context of COVID-19)? If so, how? What information? [e.g., telehealth?] How to get in contact with your provider?

3. Are you continuing to see your provider for routine visits? (think medically complex folks who may have frequent visits routinely) [Any trouble seeing your provider?]

4. Oh, you’re not going into the office. Are there other ways you are in touch with your PCP, such as telehealth options including telephone or videoconferencing visits?

- Have you used telehealth (i.e. seen the doctor over the phone/video calling)?
  - Are you using technology such as FaceTime or another smartphone app? Or a service like Zoom or Skype?
- Were you given the option to see your doctor/provider in person or having a telehealth appointment or was this choice made for you?
- How many telehealth visits have you had over the last couple weeks/month?
  - Do these meet your definition of a “good care visit”? Are you satisfied with them?
  - If you needed to, were you able to go to the office/hospital (maybe for lab tests or vaccinations)?
- Which other providers would you want to see via telehealth?
- Do you think you would want to make telehealth part of your regular care routine after Covid-19?
- Have you had any issues with telehealth, for example with internet access or having the right equipment like a smartphone? Did you prefer one method over another?

5. Can you continue to focus on your health issues (or are you "distracted" by COVID-19)?

[Information from MassHealth – reference again for their awareness]

- General eligibility and enrollment questions should go to **MassHealth CSC**:
  - Call: ([800) 841-290](tel:+18008412900)0
  - TTY: (800) 497-4648
  - Hours: Monday - Friday, 8am till 5pm
- ​Patients with COVID-19 related health questions should be directed to call their provider's office or 2-1-1 for guidance.
- In general, managed care patients with access to care issues can contact **My Ombudsman**:
  - ​Call: (855) 781-9898; VideoPhone: (339) 224-6831; TTY: use MassRelay at 711
  - Hours: Monday–Friday, 9 a.m.–4 p.m.
  - Email: [info@myombudsman.org](mailto:info@myombudsman.org)
  - Or visit: [http://www.myombudsman.org](https://nam01.safelinks.protection.outlook.com/?url=http%3A%2F%2Fwww.myombudsman.org%2F&data=02%7C01%7CAparna.Kachoria%40umassmed.edu%7C61f8d42be6694d3e44f508d7d0068ab9%7Cee9155fe2da34378a6c44405faf57b2e%7C0%7C0%7C637206598825681017&sdata=31snEXaPwtMLVBfRKnEC1jIZeqO0lGENc8y8lQOr7zE%3D&reserved=0)

Is there anything else that you would like to share with us today regarding your or your child’s experiences as a MassHealth ACO patient? **[Note to Interviewer: Please remind interviewee that they should not provide any identifying details such as dates or month of care received, names of doctors, or other identifying information.]**

Thank you for your time today.

**[Turn Recorder Off]**

To better understand patient experience, we are asking some **optional** demographic information questions. You do not have to answer any question you do not want to.

[Go through demographic form questions]

As a token of our appreciation, we will be sending you your gift card via mail or email. Which would you prefer?

[Note down email address or physical address]

Thank you again for your time. We really appreciate your perspective and input.

*** COVID-19 Questions Were Added April 2020**

**Interview Guide - MassHealth Leadership**

**Goals of the MassHealth Leadership/Staff Key Informant Interviews (KIIs):**

To obtain MassHealth Leaders/Staff perspective on delivery system reform and DSRIP program implementation (process and progress) from their particular vantage point, both internally, within MassHealth, and externally, with ACOs, CPs, and other stakeholders (e.g. health systems and patients)

To understand the goals, organizational structure and system transformation/changes built into the DSRIP program from the perspective of MassHealth leaders/staff involved in design and/or implementation; and the ways in which their interpretation of these have shaped their expectations/evaluations for process and progress.

To identify delivery system and DSRIP implementation successes and challenges, innovations, and opportunities, both inside MassHealth as well as related to the ACOs, CPs and other stakeholders.

To explore MassHealth actions to support delivery system transformation so far, and lessons learned that have informed course corrections or might inform activities over the next few years

1. **Introduction**
2. We’d like to understand your perspective on DSRIP/Demonstration implementation. What has been/is your role at Mass Health regarding DSRIP (design, development, implementation, evaluation (e.g., performance and quality indicators)? What are your responsibilities?
   1. What have been the major changes in your role, if any?
   2. How long have you worked in this role? And healthcare in general?
   3. Do you view this work through any particular lens, shaped by your current role/responsibilities or previous experiences?
   4. How do you stay up to date with the process and progress of implementing DSRIP? Meetings? Newsletters and other documents? Interactions with stakeholders? (e.g., DSRIC meetings, etc.)
3. What is MassHealth’s overarching strategy to improving care and quality while lowering costs? How is that strategy applied to the current Demonstration and DSRIP program?
   1. What are the levers MassHealth is using through the Demonstration to bring about desired changes?
      1. Where do contracting requirements, quality measures, financial risk for total cost of care, and DSRIP funds as incentives fit into the overall strategy?
      2. Are there levers outside of those four areas MassHealth is using?
4. **DSRIP Investments**
5. Overall, how have DSRIP funds been spent?
   1. At MassHealth specifically, were DSRIP funds spent mostly on furthering existing initiatives or bringing about new programs?
   2. Outside of MassHealth (at ACOs and CPs), were DSRIP funds spent mostly on furthering existing initiatives or bringing about new programs?
      1. Have DSRIP funds been spent in the way MassHealth had hoped?
6. MassHealth has undergone significant internal or intra-organizational changes in operations to implement the DSRIP program. These may have been changes in structure (e.g., the “org chart”), roles or responsibilities (“who does what”), or perhaps in “culture” (reflecting the shift from a fee-for-service to value-based payment “mentality”, population health concerns, cost containment, etc.).
   1. What have been the most important changes MassHealth has made organizationally/internally, so, far to promote health system transformation in the context of DSRIP?
      1. Successes or challenges? Facilitators or barriers? Innovations? Lessons learned?
7. **MassHealth Development and implementation of Quality and Performance Indicators**
8. Please share your perspective on MassHealth’s engagement in the process of setting and assessing performance indicators.
   1. How were certain domains of measures (e.g., for a specific patient population, for certain types of care) identified as priorities for inclusion in the ACO and CP measure slates?
   2. What was the process for choosing between existing measures and development of new measures by the state?
   3. What was the process for working with stakeholders within the state and gaining consensus on which metrics to include? What about CMS?
   4. Have there been any changes in the metrics? Why?
   5. How was the level of accountability associated with quality measure performance determined?
   6. How many performance measures and what mix of process and outcome measures were considered important for evaluating performance without overburdening ACOs and CPs?
9. What have been the challenges in operationalizing the metrics? What has been done to overcome those challenges?
   1. How did the state seek to maintain fairness in quality performance evaluation across diverse organizations with unique patient populations?
10. What are the strategies that, to you, appear to be successful in improving quality and value of healthcare?
11. **MassHealth DSRIP Activities to support ACOs**

Now let’s shift to specific DSRIP-related activities for the ACO program. Your perspective and level of insight into these will obviously vary according to your role and responsibilities, or perhaps your previous experiences. People may even differ in their definitions of these activities. But let me ask you about the following, and I can learn more about your perspective as we go along.

***Care Coordination/Integration:***

1. How do you define care coordination and service integration?
   1. What key activities have you seen, internal to MassHealth that support these activities?
   2. What new activities have you seen ACOs implement to support these activities?
   3. What is working particularly well? Or not so well? Does it vary across ACOs?
   4. What factors/conditions have worked to support innovation and improvement?
   5. Have there been challenges? How has MassHealth/organizations responded to challenges
2. Have you seen any difference around care coordination or service integration when it comes to LTSS services? How so? Or why not?
   1. What is working particularly well? Or not so well?
   2. What factors/conditions have worked to support innovation and improvement?
   3. Have there been challenges? How has MassHealth/organizations responded to challenge?
3. Keeping on the idea of integration, can you describe how the integration with Community Partners is going for the ACOs? What actions have you seen ACOs take to implement this part of DSRIP?
   1. What is working particularly well? Or not so well?
   2. What factors/conditions have worked to support innovation and improvement?
   3. Have there been challenges? How has MassHealth and/or organizations responded to challenges?
   4. What has MassHealth done to support these relationships?
   5. We know that originally ACOs had to contract with all BH CPs in their service area and at least 2 LTSS CPs. We also know there have been some discussion about changing the CP program (CP 2.0). Can you describe what MassHealth was hoping to get out of the first configuration and why they are making changes?
   6. One thing we heard a lot in some of our interviews was that how ACOs and CPs shared information and coordinated care depended a lot on the relationship the two organizations had. For CPs specifically, they often had to deal with multiple ways to send care plans or patient information to ACOs (ISS) and that was sometimes a barrier. Why did MassHealth not standardize or require any specific way to share information? Have you seen any issues around that flexible set up?

***Population Health Management***

1. Please describe what ACOs are doing for population health management and non-CP related care coordination?
   1. What are the actions MassHealth has taken to support these activities?
   2. What is working particularly well? Or not so well? Does it vary across ACOs?
   3. What factors/conditions have worked to support innovation and improvement?
   4. Have there been challenges? How has MassHealth and/or organizations responded to challenges?

***Health Related Social Needs***

1. Please describe what ACOs are doing around Health-related Social Needs (HRSN) screenings and then addressing those needs.
   1. What key activities has MassHealth taken to support these activities?
   2. What is working particularly well? Or not so well?
   3. What factors/conditions have worked to support innovation and improvement?
   4. Have there been challenges? How has MassHealth and/or organizations responded to challenges?

***Flexible Services Program***

1. Please describe how ACOs are planning to utilize the flexible services program.
   1. What key activities has MassHealth taken to support these activities?
   2. There have been several adjustments to the flexible services program since the start of the Demonstration such as scope of the program and timing of rollout. Why did these changes occur?
   3. Will these changes impact the overall success of the program?

***Data Analytics and Reporting***

1. Please describe what ACOs are doing around data analytics and data reporting.
   1. What key activities have you seen, either internal to MassHealth (to support these activities) or in the field (to implement these activities)?
   2. What is working particularly well? Or not so well?
   3. What factors/conditions have worked to support innovation and improvement? Have there been challenges?

***Health Information Technology***

1. Please describe what ACOs are doing with implementing and using Health Information Technology?
   1. What has MassHealth done to support these actions?
   2. What is working particularly well? Or not so well?
   3. What factors/conditions have worked to support innovation and improvement?
   4. Have there been challenges? How has MassHealth or organizations responded to those challenges?

***Financial Performance/Engagement***

1. How would you describe how the ACOs are doing financially? What strategies are they taking for financial performance/sustainability?
   1. What is MassHealth doing to support these actions?
   2. What is working particularly well? Or not so well?
   3. What factors/conditions have worked to support innovation and improvement? Have there been challenges? How is MassHealth organizations responding to these challenges?

***Quality Performance Indicators***

1. How are the ACOs and CPs doing with meeting their QPI metrics? What are they doing to meet those metrics?
   1. How has MassHealth supported the in those activities?
   2. What is working well and not so well?
   3. What factors contribute to success/innovation? How Is MassHealth/ organizations responding to challenges?

***Provider Engagement***

1. What does provider engagement by ACOs mean to you? What is it “supposed” to look like? How does this compare with what is happening in the field?
   1. What strategies have you heard ACOs are using to engage providers? (e.g., informative meetings, better communication, etc.)
   2. How effective are these strategies?
   3. Some ACOs, for example, are using financial incentives for primary care providers, specialists, and/or hospitals as a mechanism to engage providers in delivery system changes. How are these working out?
   4. What MassHealth strategies have been used to promote provider engagement efforts?

***Patient Engagement***

1. Patient Engagement
   1. What does patient engagement mean to you? What is it “supposed” to look like? Why is it supposed to matter?
   2. Organizations are using various strategies to promote patient engagement. What strategies have you heard about? (e.g., Advisory groups, xxx, yyy, etc.) How are these working out?
   3. What MassHealth strategies have been used to promote patient engagement efforts?
2. **MassHealth DSRIP Activities to support CPs**

Now let’s shift to specific DSRIP-related activities for the CP program. Your perspective and level of insight into these will obviously vary according to your role and responsibilities, or perhaps your previous experiences. People may even differ in their definitions of these activities. But let me ask you about the following, and I can learn more about your perspective as we go along.

1. How have the CPs done with implementing the program? What did they do? Have you seen differences between BH and LTSS CPs?
   1. What has MassHealth done to help CPs start operations and implement the program?
   2. What is working particularly well? Or not so well? Does it vary across CPs?
   3. What factors/conditions have worked to support innovation and improvement? Have there been challenges? How are MassHealth and organizations responding to challenges?
   4. Do you have any thoughts about sustainability of these efforts? Is MassHealth considering ways to support CP sustainability?

***Health Information Technology***

1. Please describe what CPs have done around implementing and using Health Information Technology
   1. What key activities have you seen, either internal to Mass Health (to support these activities) or in the field (to implement these activities)?
   2. What is working particularly well? Or not so well? Does it vary across CPs?
   3. What factors/conditions have worked to support innovation and improvement? Have there been challenges?

***Data Analytics and reporting***

1. Please describe what CPs have done around using data analytics and reporting?
   1. What key activities have you seen, either internal to MassHealth (to support these activities) or in the field (to implement these activities)?
   2. What is working particularly well? Or not so well? Does it vary across CPs?
   3. What factors/conditions have worked to support innovation and improvement? Have there been challenges?

***Patient Engagement***

1. Patient Engagement
   1. Earlier when talking about the ACOs, you described patient engagement as “x”. Is that the same definition for the CPs? What actions are the CPs doing around supporting patient engagement?
      1. How are these working out? What challenges are organizations facing and how are they responding?
      2. How has Mass Health supported these efforts? How is MassHealth responding to challenges?
   2. Originally, the CP timeline for patient engagement was 30-days but was changed to 90-days. Can you describe why MassHealth made that change? Has it been helpful?
2. **ACO and CP Alignment**
3. How have ACOs and CPs been doing in establishing relationships?
   1. Are there any notable challenges or successes in these relationships so far?
   2. Have you noticed any common barriers to establishing or continuing these relationships?
   3. How has MassHealth helped to facilitate the development of these relationships?
4. **MassHealth Support for Delivery System Transformation through Statewide Investments**

1. We know that there are various statewide investments that can be used by CPs and ACOs to implement the DSRIP program. We would like to ask you about specific ones and how they are going. What has MassHealth and participating organizations done with the SWI meant to ready participating organizations to operate as ACOs and CPs? What have been challenges? How have organizations responded to those challenges?
   1. How were the SWIs identified or determined? Why were they needed?
   2. For workforce development?
      1. How specifically did the student loan forgiveness payment program fit into this?
      2. Specific strategies for recruitment, retention, and/or training?
   3. To address ED boarding challenges?
   4. To improve accessibility for people with disabilities and people for whom English is not a primary language?
2. **STAKEHOLDER ENGAGEMENT**
3. How has MassHealth engaged stakeholders throughout the state to inform the implementation of the Demonstration and DSRIP?
   1. How has the relationship with DSRIC informed implementation?
   2. Social Services Integration Working Group (SSIWG)?
   3. Monthly meetings with advocates?
   4. Are there any concerns or recommendations that have emerged from stakeholders that you haven’t been able to address? Why not?
4. **Factors External to Mass Health**
5. What local, state, or national factors have impacted the implementation of DSRIP?
6. Are there any changes that have occurred external to the Demonstration or DSRIP that you think will influence the success of the program?

**J. Covid-19 Impacts***

1. How did Covid-19 impact the implementation of DSRIP and the overall Demonstration?
   1. How did expectations for ACOs change at all as a result of the pandemic?
   2. How did expectations for CPs change at all as a result of the pandemic?
   3. Do you expect any health systems will adjust their participation in the ACO program to take on less risk as they recover financially?
   4. Disruptions from Covid-19 may lead to worsened outcome and performance measures, causing ACO losses against predefined capitated benchmarks. How was this accounted for in the crisis response (for example, adjusting capitated rates, experimenting with temporary fee-for-service reimbursement)? How long will these crisis-era adjustments last after the pandemic? How will these emergency steps impact DSRIP implementation over the next 1, 2 years?
   5. Which emergency measures put in place by MassHealth to mitigate the impact of the pandemic may also advance aspects of the Demonstration? Which might hinder the goals of the demonstration?
   6. How do you expect reimbursement for telehealth services to change after the pandemic (e.g. parity for telehealth/in-person visits, reimbursing store-and-forward or remote patient monitoring)? How might telehealth utilization during the crisis affect plans to change relative payment rates to encourage a healthy balance of in-person vs virtual care after the pandemic?
   7. How will MassHealth support or sustain telehealth adoption after the state of emergency is lifted?
   8. How has the flexible services program been impacted by social and economic disruptions for MassHealth patients, including school closures, child care and school lunch suspensions, new unemployment/underemployment?
2. **Looking to the Future**
3. Do you have suggestions for modifying MassHealth efforts to promote delivery system transformation under the current 1115 waiver? Challenges that will have to be faced?
4. Which of the delivery system reform efforts made possible through the DSRIP funding should the state support going forward? What are your thoughts about potential funding sources if DSRIP is not renewed?
5. What one change does MassHealth have to implement in the next five years to achieve the state’s long-term healthcare goals?
6. Are there any lessons that have been learned so far by MassHealth at the organizational level that should be considered going forward or would be of interest/value to other states pursuing delivery system reform?

*** COVID-19 Questions Were Added April 2020**
